# Leaf Area and Water Content Changes after Permanent and Temporary Storage

**DOI:** 10.1371/journal.pone.0042604

**Published:** 2012-08-03

**Authors:** Kevyn J. Juneau, Catherine S. Tarasoff

**Affiliations:** School of Forest Resources and Environmental Science, Michigan Technological University, Houghton, Michigan, United States of America; DOE Pacific Northwest National Laboratory, United States of America

## Abstract

Accurate measurements of leaf morphology must be taken to develop models of ecosystem productivity and climate change projections. Once leaves are removed from a plant they begin to lose water and degrade. If specimens cannot be measured immediately after harvest, it is important to store the leaves in a manner that reduces morphological changes. If preserved specimens are used, estimates that closely match fresh measurements need to be calculated. This study examined the change in leaf area after storage treatments and developed models that can be used to more accurately estimate initial leaf area. Fresh leaf area was measured from ten plant species then stored in one of two common storage treatments. After storage, leaf area was re-measured and comparisons were made between species and growth forms. Leaf area decreased the most after permanent storage treatments and the least after temporary storage. Pressed leaves shrunk over 18% while cold storage leaves shrunk under 4%. The woody dicot growth form shrunk the least in all treatments. Shrinkage was positively correlated with initial water content and dissection index, a measure of leaf shape and complexity.

## Introduction

Leaf area and shape are two measurements commonly used to calculate ecological and physiological attributes of plant communities. Leaf area is used in calculations for leaf area index [1], [2] and specific leaf area [3]–[6]. Both of these calculations are used to model vegetation productivity and photosynthetic capacity [7]–[10]. Leaf area is also used to calculate stomatal density, a leaf characteristic that is negatively correlated with increases in atmospheric carbon dioxide (CO_2_) concentrations [11], [12]. Leaf shape, particularly complexity (i.e. lobation and dissection), is correlated with hydraulic resistance [13], leaf thermal optima [14], and has been used to determine mean annual temperature of various geographic regions [15]. In order to derive accurate values for these calculations, fresh leaves should be used [16]; however, depending on the nature of the study, analysis of fresh leaves may not be possible. There are two major cases when fresh leaves cannot be analyzed: 1) leaves are collected in the field and must be temporarily stored until they are returned to a laboratory for analysis or 2) preserved herbarium specimens are used in an analysis.

When leaves are collected in the field, temporary storage is necessary if leaves cannot be analyzed within 48 hours following harvest [16]. Cornelissen et al. [16] recommends storing leaves at low temperatures in a sealed plastic bag with a moist paper towel to minimize any changes in the leaves due to desiccation and decomposition. However, if refrigeration is not an option, Cornelissen et al. [16] further suggests storage at ambient temperature in a sealed plastic bag without a moist paper towel.

Once a leaf is removed from a plant it begins to lose water [17]. Desiccation results in lowered cellular turgor pressure, which could affect the area and shape of the leaf [18]. The amount of change is species dependent. Plants with inelastic cell walls will visibly wilt after losing 1 to 3% water content. However, plants with elastic cell walls can lose 30 to 40 percent of their water content before the leaf begins to contract and a noticeable change in appearance occurs [18]–[20]. Wilting and desiccation also permanently change the structure of cell walls by altering the cellulose micellae within cell microfibrils [19], [21]; therefore, rehydration may recover the mass of a leaf [17], but not necessarily the leaf area and shape [19]. Temporarily storing or drying leaves for preservation has the potential to adversely alter leaf area and shape and may have important ramifications when stored or preserved leaves are used to obtain data.

Researchers can assess climate change and historical atmospheric CO_2_ concentrations by measuring specific leaf area and stomatal density of leaves preserved in herbaria [11], [22], [23]. Both specific leaf area and stomatal density decrease when CO_2_ concentrations increase, so by comparing those measurements between leaves collected over a span of time, models of historical atmospheric conditions can be developed. Carbon dioxide concentrations have been increasing over the past 300 years, thus resulting in a 20–40% decrease in stomatal density and an 84% decrease in specific leaf area [11], [22]. These measurements may be inaccurate and misleading due to changes in leaf area as a result of preservation.

Herbarium leaf samples undergo a preservation process of pressing and drying [24]. Thus, changes in leaf area of plants over time may be due to the degradation of the herbarium specimens and not entirely due to increasing atmospheric CO_2_ concentrations [22]. Depending on the species, effects of preservation can range from little to no effect to large reductions in size. For example, leaf area decreases ranging from 20 to 31% have been observed in various rainforest (H. Romero, personal communication) and temperate species (T. Nuttle, personal communication) after preservation. Decreases in leaf size caused by drying can overestimate the stomatal density measurements of preserved leaves. As a consequence of drying and subsequent shrinkage, researchers concluded that the stomatal density of *Pinus taeda* needles was inflated by 14% [25].

Because the extent of leaf area changes due to various preservation and storage treatments is unknown, researchers may completely disregard any differences and combine measurements from both fresh and preserved samples in their models. For example, Kouwenberg et al. [12] determined there was a correlation between atmospheric CO_2_ concentration and stomatal density and frequency. To develop their model, Kouwenberg et al. [12] used both fresh and preserved conifer leaves, without accounting for any differences between the two. This could lead to erroneous models of atmospheric CO_2_ and climate change projections. Thus, it is imperative that researchers understand and account for the potential error associated with leaf storage procedures.

This study aimed to investigate the changes in leaf area that occur after common leaf storage practices, permanently pressed dry and temporally placed into cold storage, as a function of water content and leaf complexity.

## Materials and Methods

### Species used and image analyses

Fully expanded leaves were collected from ten species of vascular plants in Houghton, Michigan, USA (Table 1). All specimens were collected from 10 to 15 September 2010, except for the conifers, *Pinus strobus* and *Picea pungens*, and the fern, *Polypodium virginianum* which were collected on 20 and 21 June 2011, respectively. Seventy-five leaves of each of species were collected from >50 individuals. Collection followed the protocol developed by Cornelissen et al. [16]; only fully expanded, hardened leaves with no signs of herbivory were selected from areas on the plant that were located in full light. Broadleaf laminas and petioles, if present, and needles were weighed with an analytical balance (American Weigh Scales, Inc. Norcross, GA). Leaves were then scanned into digital format using an Epson Expression 10000XL flatbed color image scanner (Seiko Epson Corporation, Nagano, Japan) and saved as 1200 dpi, 24-bit color, uncompressed TIFF files within 60 minutes of harvest. A weight was placed on the scanner cover to ensure the leaves were pressed firmly against the glass. Images were manipulated when necessary to remove shadows and to darken light areas on the leaves then converted to 8-bit gray scale images. All image alterations were performed using the GNU image manipulation program (GIMP) 2.6.7 (Free Software Foundation, Inc. Boston, MA). Images were then digitally analyzed for projected leaf area and perimeter using ImageJ 1.44j (National Institutes of Health, Bethesda, MD). This method of image analysis is known to be accurate [26], [27] and was used in studies to determine specific leaf area [5], leaf herbivory [28], [29], and leaf shape and structural diversity [14], [30].

**Table 1:** The ten species analyzed; their scientific and common names, growth form and initial percent water content.

Species	Abbreviation	Common name	Growth form	Water content (%)
*Acer saccharum* Marsh.	acsa	sugar maple	woody dicot	52.89±0.64
*Asclepias syriaca* L.	assy	common milkweed	forb	75.91±0.53
*Avena sativa* L.	avsa	common oat	graminoid	80.73±.99
*Phragmites australis* (Cav.) Trin. ex Steud.	phau	common reed	graminoid	61.00±1.19
*Picea pungens* Engelm.	pipu	Colorado blue spruce	conifer	61.82±3.01
*Pinus strobus* L.	pist	eastern white pine	conifer	40.19±0.55
*Pteridium aquilinum* (L.) Kuhn	ptaq	common bracken	fern	64.09±0.65
*Quercus rubra* L.	quru	northern red oak	woody dicot	52.03±0.72
*Taraxacum officinale* F.H. Wigg.	taof	common dandelion	forb	83.31±0.58
*Polypodium virginianum* L.	povi	common polypody	fern	74.21±1.47

Nomenclature follows USDA, NRCS [52]. Water content (±1 SE) was determined by averaging the percent difference between fresh mass and oven dried mass of 25 leaves for each species.

Twenty-five leaves of each species were then randomly assigned to one of two storage treatments: 1) pressed for 30 days (pressed) or 2) sealed in a 3.7 L plastic bag and refrigerated at cold temperature (2°C) for 30 days (cold storage). Air was pressed out of the plastic bags prior to sealing them, and leaves were stored in darkness. Treatment 1 is a common long-term storage method and treatment 2 represents temporary storage methods. Though leaves typically would not be temporarily stored for 30 days prior to processing, we wanted to test 30 days in cold storage as an extreme. After treatment, leaves were reweighed and rescanned. The remaining twenty-five leaves of each species were used to estimate percent water content (Table 1). The leaf initial water content for each species was calculated as the average percent difference between fresh mass and oven dried mass divided by the fresh mass (Eq. 1).

### Statistical analyses

Each species was analyzed separately using paired t-tests to compare changes between fresh and stored leaf areas. Percent change (Eq. 1) in area and relative water loss were calculated for each leaf and analysis of variance (ANOVA) was conducted to compare between functional groups in each treatment.


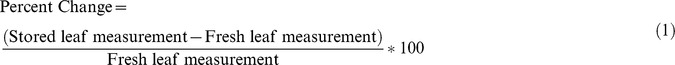


All the species were then combined and multiple regression analysis was used to determine the effects of leaf dissection index, a metric of leaf shape and complexity, and initial water content on percent change in area for each treatment.

Fresh leaf area and perimeter data obtained from the image analyses were used to determine dissection index [31]–[33]. Dissection index (Eq. 2) is a standardized metric using perimeter and the square root of an object's area to determine shape complexity; a circle has a dissection index of 1.0 while more complex shapes have higher indices.


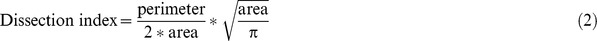


Tukey's HSD test was used when multiple comparisons were made. All assumptions of normality and homoscedastic variance of these data were met. Analyses were conducted in JMP 8.0 (SAS Institute Inc. Cary, NC).

*Ethics statement:* No specific permits were required for plant collection in this study and all plant specimens were obtained from public, university owned land; therefore, no specific permissions were required for collection. None of the plants used in this study are threatened or endangered in the state of Michigan.

## Results

### Pressed leaves

**Figure 1:**
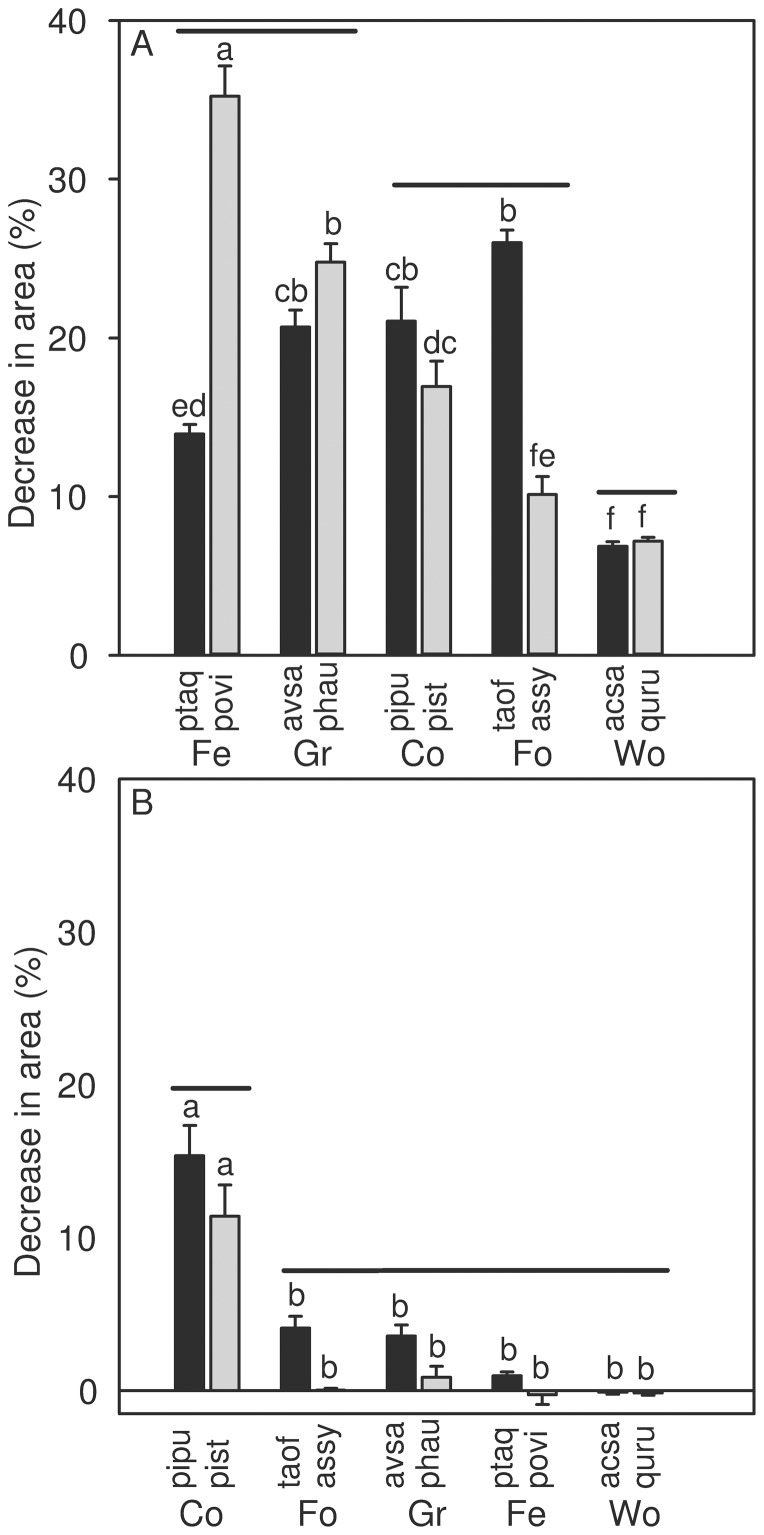
Decrease in area of different growth forms and species after two storage methods. Decrease in leaf area after (A) pressed dry and (B) cold (2°C) storage. Different letters indicate significant differences between species (P≤0.05, n = 250). Growth forms not connected by solid bars are significantly different (P≤0.05). Abbreviations for growth forms are Co - conifer, Fe - fern, Fo - forb, Gr - graminoid, Wo - woody dicot. Abbreviations for species are given in Table 1. Error bars indicate 1 SE.

All pressed leaves lost 100% of their water content during this treatment, thus resulting in significant decreases in area for all the species tested (P<0.05; Table 2). *Acer saccharum* and *Quercus rubra*, shrunk the least when pressed dry, 6.86±0.29% (mean ± 1 SE) and 7.18±0.25% respectively. *Polypodium virginianum* and *Taraxacum officinale* shrunk the most, 35.22±1.92 and 25.99±0.79% respectively. When species were nested within growth form, the woody dicots shrunk the least, 7.02±0.19%, and were statistically different than the other growth forms. The fern growth form shrunk the most 24.58±1.82% but was not significantly different than the graminoids, which shrunk 22.68±0.84% (Figure 1). On average, pressed leaves shrunk 18.25±0.67%.

**Table 2:** Difference in leaf area between fresh and treated leaves of ten species and the respective percent decrease in area.

Species	Treatment	Difference in leaf area (cm^2^)		Decrease in area (%)
*Acer saccharum*	Pressed	−3.94±0.37	*	6.86±0.29
	Cold	0.03±0.06		−0.09±0.13
*Asclepias syriaca*	Pressed	−5.87±0.56	*	10.13±1.13
	Cold	−0.09±0.07		0.05±0.10
*Avena sativa*	Pressed	−4.43±0.38	*	20.68±1.08
	Cold	−0.82±0.17	*	3.58±0.73
*Phragmites australis*	Pressed	−7.07±0.93	*	24.76±1.17
	Cold	−0.31±0.20		0.87±0.73
*Picea pungens*	Pressed	−0.08±0.01	*	21.06±2.12
	Cold	−0.05±0.01	*	15.37±1.97
*Pinus strobus*	Pressed	−0.08±0.01	*	16.94±1.58
	Cold	−0.06±0.01	*	11.41±2.06
*Polypodium virginianum*	Pressed	−8.78±0.69	*	35.22±1.92
	Cold	0.03±0.15		−0.25±0.65
*Pteridium aquilinum*	Pressed	−5.07±0.50	*	13.94±0.59
	Cold	−0.35±0.10	*	0.98±0.26
*Quercus rubra*	Pressed	−4.82±0.42	*	7.18±0.25
	Cold	0.03±0.05		−0.14±0.15
*Taraxacum officinale*	Pressed	−4.74±0.29	*	25.99±0.79
	Cold	−0.92±0.18	*	4.11±0.77

Asterisks denote differences between fresh and treated leaf areas that were significantly different than zero (paired t-test, P≤0.05, n = 25).

Both initial water content and dissection index were positively correlated with decreases in area when leaves were pressed dry, and there were no interactions between the two variables (P = 0.27). This relationship was explained by the model Δarea(%) = −14.69+0.37 percent initial water content+2.64 dissection index (R^2^ = 0.30, P<0.0001), Leaves with high initial water content and higher dissection indices shrunk more when pressed than those that did not.

### Cold storage

**Figure 2:**
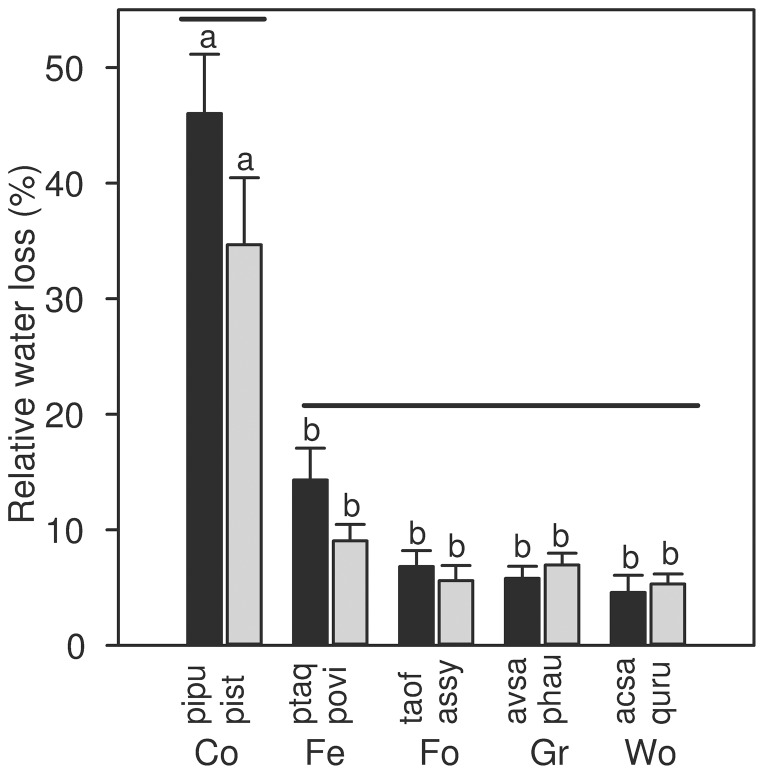
Water loss of the different growth forms and species after cold (2°C) storage. Different letters indicate significant differences within storage treatment (P≤0.05, n = 175 for ambient temperature storage, n = 225 for the other treatments). Growth forms not connected by solid bars are significantly different (P≤0.05). Abbreviations for growth forms are Co - conifer, Fe - fern, Fo - forb, Gr - graminoid, Wo - woody dicot. Abbreviations for species are given in Table 1. Error bars indicate 1 SE.

The water lost by leaves during the 30 day cold storage treatment was variable (Figure 2), resulting in an average decrease in area of 3.59±0.46% across all species. The conifers lost the most relative water content, 40.34±3.92%. The other growth forms lost between 5 and 12% water content, with the woody dicots losing the least.

Cold storage resulted in significant area decreases for *Avena sativa*, *Picea pungens*, *Pinus strobus*, *Pteridium aquilinu*, and *Taraxacum officinale* (P<0.05; Table 2). Both *Avena sativa* and *Taraxacum officinale* had the smallest significant decreases in area after cold storage, 3.58±0.73% and 4.11±0.77% respectively. *Picea pungens* displayed the largest significant shrinkage, 15.37±1.97%, followed by *Pinus strobus*, 11.41±2.06%. The conifer growth form shrunk the most, 13.38±1.44%, and was statistically different than all the other growth forms (P<0.0001; Figure 1).

Initial water content affected the decrease in area of leaves stored in cold temperatures for 30 days (P = 0.02), while dissection index did not (P = 0.96); however, there was an interaction between these two variables (P<0.0001). After cold storage, leaves with high dissection index and low initial water content shrunk the most, while leaves with a high dissection index and high initial water content increased in size. These data were also quite variable, and the model, Δarea(%) = 8.36+−0.08 initial water content+−0.02 dissection index+−0.09 initial water content* dissection index explained only 10% of the variation.

## Discussion

Results from this study indicate that storing leaves permanently or temporarily will result in considerable leaf shrinkage. When leaves are processed for long-term preservation or storage, water is removed from leaves. As leaves lose water the cellular turgor pressure decreases and the cell membrane separates from the cell wall [34]. After a critical point is reached the cell wall collapses resulting in deformations, folds, and subsequent shrinkage of the leaf [18]. For example, desiccation of *Craterostigma wilmsii* results in a 78% decrease in cell volume due to cell wall collapse [35]. Pressing, which forces the leaves to hold their shape while drying, thus preventing wilting and crimpling, will not prevent a leaf from shrinking. Though not tested for in this study, there may be a possibility to return pressed leaves to their original area through rehydration if pressed leaves are dried at low temperatures. Drying leaves at low temperatures would reduce the breakdown of the structural cellulose in the leaves because hydrogen bonds, which form the cellulose polymers in the cell walls, are altered at temperatures above 40°C [36]. Alteration to these bonds at high temperatures results in weakened, brittle leaves. Cell walls are a matrix of molecules and contain 60% water [37], so removal of this water at high temperatures will also result in a less fluid, more rigid molecular complex, and would be less likely to return to their original area and shape through rehydration.

Cold temperatures are known to reduce microbial activity and decomposition [38]; therefore, cold storage at 2 to 3°C is the recommended temporary storage procedure [16]. Leaves stored for 30 days at 2°C in this study showed no observable signs of degradation. In addition to a decrease in decomposition rates, cold temperatures provided less energy for water to evaporate out of leaves through diffusion and air movement [39]. Reduced evaporation rates helped maintain the cell turgor pressure during storage [18], and thus sustained the leaf areas. The conifers did, however, lose the most water and shrank drastically after cold storage. The decrease in conifer area may be amplified because of their small initial area; however, the shrinkage may also be the consequence of xylem collapse, a phenomenon that occur in conifers, particularly in the tracheid cells that deliver water from the midrib to the leaf margins [40], [41]. Xylem collapse is a mechanical process that is thought to be an adaption to prevent cavitation by reducing the diameter of the xylem to maintain sap flow [40], [41]. The flattening of xylem in the leaf tissue would pull the leaf margin inward, reducing the width of the leaf and consequently its area. Xylem collapse will occur even if surrounding phloem and parenchyma cells remain hydrated and turgid [40].

While water loss during storage in important, it is not the only factor affecting leaf shrinkage. Both initial water content and dissection index influenced changes in leaf area. Initial water content is important because plants with high initial water content rely more on turgor pressure for support than do leaves with low initial water content, especially those with thin cell walls [42], [43]. Leaves with high dissection indices also shrank the most when pressed. Leaves with complex shapes typically have mesophyll tissue supplied primarily with first- and second-order veins while less complex leaves have more high-order venation [44]. The additional high-order venation throughout the leaf tissue would provide additional structural support to the leaf and reduce the amount of shrinkage, especially if the xylem is heavily lignified.

Though not investigated in this study, we expect other variables could help explain more of the variation associated with leaf shrinkage. One possible explanation for the small change in area for woody dicot species and large changes for forbs and grasses could be the lignin content of these growth forms. One of the functions of lignin is structural support [45]. The growth forms used in this study have lignin contents that range from high, >30% in ferns [46] and 22% to 26% in the woody dicots and conifers [47]–[49], to as low as 5% for the grasses [50], [51]. We would expect that the higher lignin content would prevent leaf shrinkage, which appears to be occurring except in the fern growth form.

### Conclusions

This study has shown that both temporary and permanent storage will result in the significant shrinkage of leaves. Temporarily stored leaves must be refrigerated and should be processed immediately to prevent large area decreases. If measurements are taken from samples preserved for herbaria, we recommend using growth forms that result in the least amount of shrinkage. Because shrinkage does occur, it must be accounted for. If not, calculations for specific leaf area, leaf area index, stomatal density, or any other area-dependant calculations may not be accurate. The results of this study shed light on an issue that has gone undocumented in much of the literature. By correcting for the decrease in area of stored leaves, more accurate projections and models can be made for both ecological processes and climate projections.

## References

[1] Watson DJ (1947) Comparative physiological studies on the growth of field crops: I. Variation in net assimilation rate and leaf area between species and varieties, and within and between years. Ann Bot 11: 41–76.

[2] Bréda NJ (2003) Ground-based measurements of leaf area index: a review of methods, instruments and current controversies. J Exp Bot 54: 2403–2417. 14565947 10.1093/jxb/erg263

[3] Evans J, Poorter H (2001) Photosynthetic acclimation of plants to growth irradiance: the relative importance of specific leaf area and nitrogen partitioning in maximizing carbon gain. Plant Cell Environ 24: 755–767.

[4] Garnier E, Shipley B, Roumet C, Laurent G (2001) A standardized protocol for the determination of specific leaf area and leaf dry matter content. Funct Ecol 15: 688–695.

[5] Bond-Lamberty B, Wang C, Gower S, Norman J (2002) Leaf area dynamics of a boreal black spruce fire chronosequence. Tree Physiol 22: 993–1001. 12359526 10.1093/treephys/22.14.993

[6] Li Y, Johnson DA, Su Y, Cui J, Zhang T (2005) Specific leaf area and leaf dry matter content of plants growing in sand dunes. Bot Bull Acad Sin 46: 127–134.

[7] Prado CH, De Moraes J (1997) Photosynthetic capacity and specific leaf mass in twenty woody species of Cerrado vegetation under field conditions. Photosynthetica 33: 103–112.

[8] Reich PB, Walters MB, Ellsworth DS (1997) From tropics to tundra: global convergence in plant functioning. Proc Natl Acad Sci 94: 13730–13734. 9391094 10.1073/pnas.94.25.13730 PMC28374

[9] Reich PB, Ellsworth DS, Walters MB (1998) Leaf structure (specific leaf area) modulates photosynthesis—nitrogen relations: evidence from within and across species and functional groups. Funct Ecol 12: 948–958.

[10] Reich PB, Ellsworth DS, Walters MB, Vose JM, Gresham C, et al (1999) Generality of leaf trait relationships: a test across six biomes. Ecology 80: 1955–1969.

[11] Woodward F (1987) Stomatal numbers are sensitive to increases in CO_2_ from pre-industrial levels. Nature 327: 617–618.

[12] Kouwenberg LLR, McElwain JC, Kurschner WM, Wagner F, Beerling DJ, et al (2003) Stomatal frequency adjustment of four conifer species to historical changes in atmospheric CO2. Am J Bot 90: 610–619. 21659156 10.3732/ajb.90.4.610

[13] Sisó S, Camarero J, Gil-Pelegrín E (2001) Relationship between hydraulic resistance and leaf morphology in broadleaf Quercus species: a new interpretation of leaf lobation. Trees-Struct Funct 15: 341–345.

[14] Nicotra A, Cosgrove M, Cowling A, Schlichting C, Jones C (2008) Leaf shape linked to photosynthetic rates and temperature optima in South African Pelargonium species. Oecologia 154: 625–635. 17943318 10.1007/s00442-007-0865-1

[15] Royer DL, Wilf P, Janesko DA, Kowalski EA, Dilcher DL (2005) Correlations of climate and plant ecology to leaf size and shape: potential proxies for the fossil record. Am J Bot 92: 1141–1151. 21646136 10.3732/ajb.92.7.1141

[16] Cornelissen J, Lavorel S, Garnier E, Diaz S, Buchmann N, et al (2003) A handbook of protocols for standardised and easy measurement of plant functional traits worldwide. Aust J Bot 51: 335–380.

[17] Ryser P, Bernardi J, Merla A (2008) Determination of leaf fresh mass after storage between moist paper towels: constraints and reliability of the method. J Exp Bot 59: 2461–2467. 18469322 10.1093/jxb/ern120 PMC2423665

[18] Kramer PJ, Boyer JS (1995) Water relations of plants and soils. San Diego CA: Academic Press. 495.

[19] Weatherley P (1965) The state and movement of water in the leaf. Symp Soc Exp Biol 19: 157–184. 5321563

[20] Evans GC (1972) The quantitative analysis of plant growth. Berkley CA: University of California Press. 734.

[21] Milthorpe F, Spencer E (1957) Experimental studies of the factors controlling transpiration. J Exp Bot 8: 413–437.

[22] Peñuelas J, Matamala R (1990) Changes in N and S leaf content, stomatal density and specific leaf area of 14 plant species during the last three centuries of CO_2_ increase. J Exp Bot 41: 1119–1124.

[23] Miller-Rushing AJ, Primack RB, Templer PH, Rathbone S, Mukunda S (2009) Long-term relationships among atmospheric CO2, stomata, and intrinsic water use efficiency in individual trees. Am J Bot 96: 1779–1786. 21622298 10.3732/ajb.0800410

[24] Hicks AJ, Hicks PM (1978) A selected bibliography of plant collection and herbarium curation. Taxon 27: 63–99.

[25] Hultine KR, Marshall JD (2001) A comparison of three methods for determining the stomatal density of pine needles. J Exp Bot 52: 369–373. 11283182

[26] O'Neal ME, Landis DA, Isaacs R (2002) An inexpensive, accurate method for measuring leaf area and defoliation through digital image analysis. J Econ Entomol 95: 1190–1194. 12539831 10.1603/0022-0493-95.6.1190

[27] Abràmoff MD, Magalhães PJ, Ram SJ (2004) Image processing with ImageJ. Biophotonics International 11: 36–42.

[28] McAuslane HJ, Alborn HT (2000) Influence of previous herbivory on behavior and development of Spodoptera exigua larvae on glanded and glandless cotton. Entomol Exp Appl 97: 283–291.

[29] Wheeler DA, Isman MB (2001) Antifeedant and toxic activity of Trichilia americana extract against the larvae of Spodoptera litura. Entomol Exp Appl 98: 9–16.

[30] Sack L, Frole K (2006) Leaf structural diversity is related to hydraulic capacity in tropical rain forest trees. Ecology 87: 483–491. 16637372 10.1890/05-0710

[31] Kincaid DT, Schneider RB (1983) Quantification of leaf shape with a microcomputer and Fourier transform. Can J Botany 61: 2333–2342.

[32] McLellan T (1993) The roles of heterochrony and heteroblasty in the diversification of leaf shapes in Begonia dregei (Begoniaceae). Am J Bot 80: 796–804.

[33] McLellan T, Endler JA (1998) The relative success of some methods for measuring and describing the shape of complex objects. Syst Biol 47: 264–281.

[34] Vicré M, Lerouxel O, Farrant J, Lerouge P, Driouich A (2004) Composition and desiccation-induced alterations of the cell wall in the resurrection plant Craterostigma wilmsii. Physiol Plantarum 120: 229–239. 10.1111/j.0031-9317.2004.0234.x 15032857

[35] Farrant JM (2000) A comparison of mechanisms of desiccation tolerance among three angiosperm resurrection plant species. Plant Ecol 151: 29–39.

[36] Watanabe A, Morita S, Ozaki Y (2006) Temperature-dependent structural changes in hydrogen bonds in microcrystalline cellulose studied by infrared and near-infrared spectroscopy with perturbation-correlation moving-window two-dimensional correlation analysis. App. Spectrosc 60: 611–618. 10.1366/000370206777670549 16808862

[37] Brett CT, Waldron K(1996) Physiology and biochemistry of plant cell walls. London: Chapman and Hall. 255.

[38] Kätterer T, Reichstein M, Andrén O, Lomander A (1998) Temperature dependence of organic matter decomposition: a critical review using literature data analyzed with different models. Bio Fert Soils 27: 258–262.

[39] Gates DM (1968) Transpiration and leaf temperature. Ann Rev of Plant Physio 19: 211–238.

[40] Cochard H, Froux F, Mayr S, Coutand C (2004) Xylem wall collapse in water-stressed pine needles. Plant Physiol 134: 401–408. 14657404 10.1104/pp.103.028357 PMC316319

[41] Brodribb TJ, Holbrook NM (2005) Water stress deforms tracheids peripheral to the leaf vein of a tropical conifer. Plant Physiol 137: 1139–1146. 15734905 10.1104/pp.104.058156 PMC1065413

[42] Niklas KJ (1986) Biomechanical responses of chive (Allium schoenoprasum var. shoenoprasum) leaves to changes in water potential. Am J Bot 73: 636–637.

[43] Niklas KJ (1989) Mechanical behavior of plant tissues as inferred from the theory of pressurized cellular solids. Am J Bot 76: 929–937.

[44] Holbrook NM, Zwieniecki MA(2005) Vascular transport in plants. Burlington MA: Elsevier Academic Press. 564.

[45] Campbell MM, Sederoff RR (1996) Variation in Lignin Content and Composition (Mechanisms of Control and Implications for the Genetic Improvement of Plants). Plant Physiol 110: 3–13. 12226169 10.1104/pp.110.1.3 PMC157688

[46] Robinson JM (1990) Lignin, land plants, and fungi: biological evolution affecting Phanerozoic oxygen balance. Geology 18: 607–610.

[47] White RH (1987) Effect of lignin content and extractives on the higher heating value of wood. Wood Fiber Sci 19: 446–452.

[48] Harmon ME, Baker GA, Spycher G, Greene SE (1990) Leaf-litter decomposition in the Picea/Tsuga forests of Olympic National Park, Washington, USA. Forest Ecol Manag 31: 55–66.

[49] Gessner MO, Chauvet E (1994) Importance of stream microfungi in controlling breakdown rates of leaf litter. Ecology 75: 1807–1817.

[50] Van Soest PJ (1994) Nutritional ecology of the ruminant. Ithaca NY: Cornell University Press. 476.

[51] Schaefer D, Steinberger Y, Whitford WG (1985) The failure of nitrogen and lignin control of decomposition in a North American desert. Oecologia 65: 382–386. 28310443 10.1007/BF00378913

[52] USDA, NRCS (2011) The PLANTS Database. National Plant Data Team, Greensboro, NC Available: http://plants.usda.gov. Accessed December 31, 2011.

